# Bone metabolism and clinical study of 44 patients with bisphosphonate-related osteonecrosis of the jaws

**DOI:** 10.4317/medoral.17946

**Published:** 2012-08-28

**Authors:** María S. Bocanegra-Pérez, Mario Vicente-Barrero, Manuel Sosa-Henríquez, Eduardo Rodríguez-Bocanegra, José M. Limiñana-Cañal, Ariadna López-Márquez, Daniel Pérez-Plasencia, Angel Ramos-Macías

**Affiliations:** 1 Medical Doctor. Stomatologist. Insular Maternal and Child University Hospital Complex of Las Palmas of Gran Canaria. Stomatology, Oral and Maxillofacial Surgery Departments. Bone Metabolism Unit and Research Unit. Las Palmas of Gran Canaria, Spain; 2 Medical Doctor. Stomatologist. Associate Professor. Insular Maternal and Child University Hospital Complex of Las Palmas of Gran Canaria. Stomatology, Oral and Maxillofacial Surgery Departments. Bone Metabolism Unit and Research Unit. Las Palmas of Gran Canaria, Spain; 3 Medical Doctor. Professor in General Pathology. Insular Maternal and Child University Hospital Complex of Las Palmas of Gran Canaria. Stomatology, Oral and Maxillofacial Surgery Departments. Bone Metabolism Unit and Research Unit. Las Palmas of Gran Canaria, Spain; 4 Biology Student. Insular Maternal and Child University Hospital Complex of Las Palmas of Gran Canaria. Stomatology, Oral and Maxillofacial Surgery Departments. Bone Metabolism Unit and Research Unit. Las Palmas of Gran Canaria, Spain; 5 Research Unit. Professor in Statistics. Insular Maternal and Child University Hospital Complex of Las Palmas of Gran Canaria. Stomatology, Oral and Maxillofacial Surgery Departments. Bone Metabolism Unit and Research Unit. Las Palmas of Gran Canaria, Spain; 6 Odontologist. Insular Maternal and Child University Hospital Complex of Las Palmas of Gran Canaria. Stomatology, Oral and Maxillofacial Surgery Departments. Bone Metabolism Unit and Research Unit. Las Palmas of Gran Canaria, Spain; 7 Medical Doctor. Otorhinolaryngologist. Associate Professor. Insular Maternal and Child University Hospital Complex of Las Palmas of Gran Canaria. Stomatology, Oral and Maxillofacial Surgery Departments. Bone Metabolism Unit and Research Unit. Las Palmas of Gran Canaria, Spain; 8 Medical Doctor. Otorhinolaryngologist. Tenured Professor. Insular Maternal and Child University Hospital Complex of Las Palmas of Gran Canaria. Stomatology, Oral and Maxillofacial Surgery Departments. Bone Metabolism Unit and Research Unit. Las Palmas of Gran Canaria, Spain

## Abstract

Osteonecrosis of the jaws is a clinical entity described and linked to treatment with bisphosphonates in 2003. Its real incidence is unknown and it could increase due to the large number of patients treated with these drugs, and its cumulative effect on the bone. State of the art knowledge regarding its etiopathogeny, clinical course and suitable treatments is limited. Objectives: To study the clinical characteristics of 44 patients with bisphosphonate-related osteonecrosis of the jaws and the state of their bone mineral metabolism: bone remodeling state, prevalence of fractures, bone mineral density study, and assessment of the different treatment strategies. Design of the Study: Observational. Information was gathered prospectively through interviews, clinical examinations, additional tests and review of medical records. Results: We studied 16 men and 28 women with a mean age of 64.7 years. Breast cancer was the most frequent underlying disease. Zoledronate was used in 82% of the cases and in the non-oncology group of patients; alendronate was the most frequently used bisphosphonate. The mean duration of the zoledronate and alendronate treatments was 25 months and 88 months respectively. The lower jaw was the most frequent location, and previous exodontias—among the triggering factors known—were the most closely linked to its onset. We found considerable osteoblastic activity in patients suffering from neoplasia, with artifacts present in their bone densitometry and a high percentage of vertebral fractures. Conclusions: According to our results, osteonecrosis of the jaws affects elderly patients. We found a direct relationship between the duration of exposure and the accumulated dose. Other relevant factors are: Poor oral and dental health, corticoids, diabetes and teeth extractions. In essence, it is a clinical diagnosis. Prevention is the best strategy to handle this clinical entity.

** Key words:**Alendronate, bisphosphonate, jaw, maxilla, osteonecrosis, osteoporosis, prevention, zoledronate.

## Introduction

Bisphosphonates (BPs) are highly effective drugs to treat conditions associated to bone resorption increases—these drugs were initially used for Paget’s disease and then for malignant hypercalcemia (MHC), multiple myeloma (MM), bone metastasis and osteoporosis.

Bone in general is considered to be the third metastasis location. Using BPs—administered intravenously (IV) into the tumor bone diseased—is a leap forward taken in the last few years (1,2). Recently, using zoledronic acid in an annual, single dose has been added to the treatment of postmenopausal osteoporosis (3).

Oral BPs are the most thoroughly studied drugs to prevent bone loss and reduce fractures. Bone Mineral Density (BMO) in-creases mainly in the lumbar spine, but also in the proximal femur (4,5).

BPs are well tolerated if administered correctly. In the last few years, a complication that had not been detected in previous clinical trials has been described: Osteonecrosis of the jaws (ONJ), which seems to be due to a combination of lack of vascular supply, lack of bone remodeling and regeneration (6).

Its onset has been associated to many risk factors. The type, the administered dose and the administration route of the BP (7) have an impact. Duration of treatment is an important factor as well, as are the presence of repeated traumatisms in the buccal mucosa, or prior dentoalveolar surgery (8-10). Additionally, diabetic patients, patients suffering from peripheral vascular diseases, smokers, and patients receiving corticoids and/or anti-neoplasic treatments concomitantly should be examined in greater detail (7,10).

## Material and Methods

This study assesses all patients referred to the Stomatology, Oral and Maxillofacial Surgery Department of the Insular Maternal and Child University Hospital Complex between March 2007 and December 2010, who were potentially suffering from BP-related ONJ, or presenting a dental pathology and long-term BP treatment. We examined 70 patients and confirmed the ONJ diagnosis in 44 cases, which we selected as sample for our observational study.

We applied the AAOMS inclusion criteria (11): 1. Current or prior BP treatment; 2. Presence of exposed necrotic bone in the maxillofacial region for more than 8 weeks; 3. No radiotherapy to the jaws; later modified by Bagán (12) to include the presence of fistulas, even without exposed necrotic bone, as an incipient ONJ stage. Patients with maxillary neoplasia were excluded.

After obtaining their consent for the study, patients answered to a general and dental health questionnaire, specially focused on risk factors, diseases and medications that could affect bone mineral metabolism.

Analytics were requested with a focus on osteoporosis: hip and spine densitometry (DXA) and ultrasound of the calcaneus (QUS). Salivary pH was measured with Macherey-Nagel ® test paper, the prevalence of cavities with the DMF index, gum’s health with the gingival index (GI), and the patient’s ability to remove bacterial plaque with the Patient Hygiene Performance index (PHP). An orthopantomography and pictures were taken, and additional tests were requested as needed (culture and/or biopsy, lateral spine radiographies of the dorsal-lumbar areas, bone gammagraphy and/or maxillary computerized tomography scan). Lastly, they were recommended pharmacological treatment, and based on the size and condition of their lesions, daily local cleansings were recommended, or other necessary treatments. They were given advice on oral and dental hygiene, healthy habits and recommendations on the dental, periodontal or prosthetic treatment they needed.

Data were included in a Microsoft Excel ® spreadsheet and then imported to the statistical software SPSS ® (Statistical Package for the Social Sciences) for Windows, version 17. Seeking to compare some results, we divided our patients into three groups based on their underlying conditions (neoplasia, multiple myeloma or osteoporosis with rheumatoid arthritis); although we did not seek to obtain conclusive data, having disparate groups and a small sample size. The SNK (Student-Newman-Keuls) multiple comparison test was performed.

## Results

This study includes 44 patients in total, 16 men and 28 women ( Table 1).

**Table 1:**
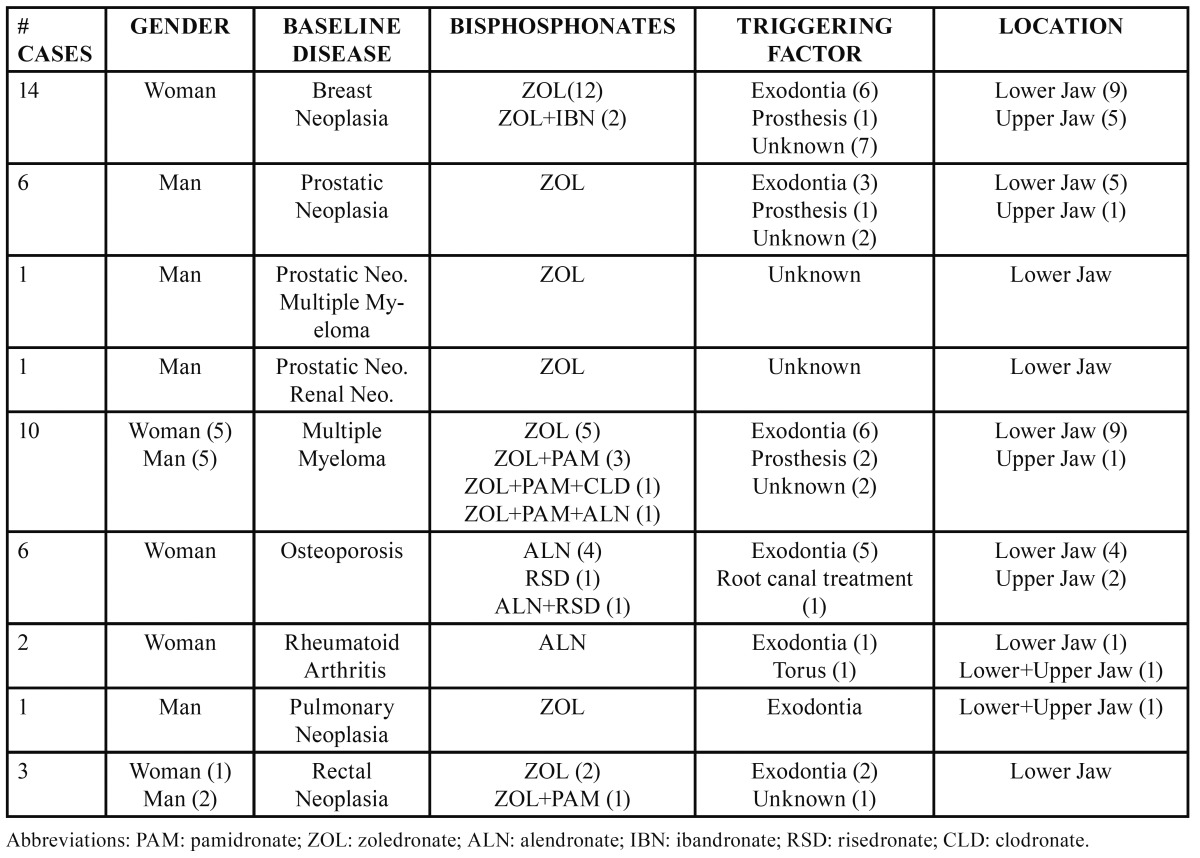
Presentation of Clinical Cases.

Among the anthropometric baseline characteristics, the mean age, 64.2 years (41-82 years), is noteworthy. Regarding habits and lifestyle, 22% of the sample used to smoke (15 cigarettes/day on average for 28 years on ave-rage) and 27% were ex smokers. We did not find regular intake of alcohol. Additionally, 52% did not exercise at all. Besides, 64% of patients had a daily calcium intake below 1200 mg; 55% of patients took calcium supplements, with or without vitamin D. And 66% brushed their teeth more than once per day, whereas 21% did not brush their teeth. The most frequent concomitant diseases found were arterial hypertension (45%) and type II diabetes (35%). The most frequent concomitant treatments found were: Chemotherapy (81%), gastric protection (75%), tranquilizers (68%), corticoids (66%) and hormonal therapy (51%).

Zoledronate was used in 36 cases (82%) and in the non-oncology group of patients; alendronate was the most frequently used bisphosphonate (87.5%). The average duration of the zoledronate treatment was 25 months (7-51 months); and of the alendronate treatment, 88 months (32-176 months). The total dose taken by patients treated only with zoledronate was 102 mg on average (36-204 mg), whereas those patients receiving only alendronate took 25,428 mg on average (8,960-51,970 mg). After confirming the ONJ diagnosis, the BP treatment was stopped. We have found a triggering factor in all cases related to oral BPs. Broadly, the triggering factor is unknown in 32% of the sample, and related to previous exodontias in 54.5% of the cases ( Table 1).

Lesions were located in the lower jaw in 75% of the cases ( Table 1), and duration-wise, the longest case is found in the superior maxilla—5.7 years at the conclusion of the study.

No patient had a full dentition, being the average DMF teeth value 13 in women, and 10 in men. They presented poor conservative dental treatment (16%), and widespread periodontal disease. The average GI in women was 1.9, and in men, 1.3; and the average PHP in women was 25%, and in men, 30%. Salivary pH was 6.5 on average.

**Figure 1:**
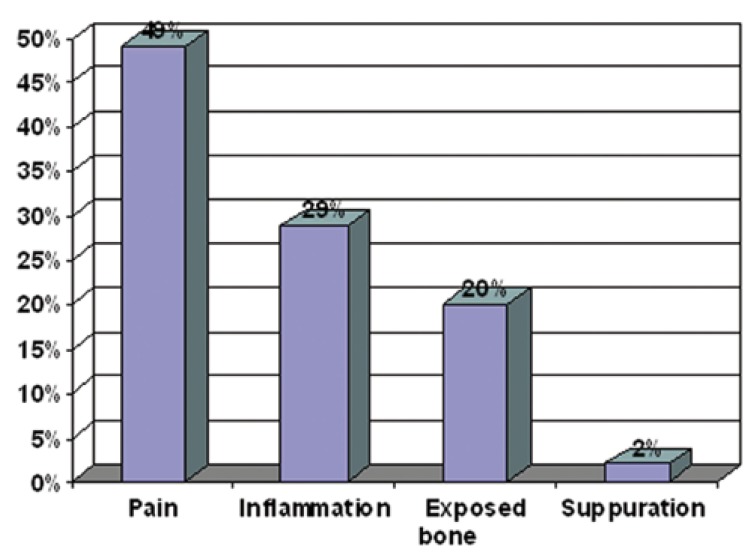
First Symptom or Onset of this Pathology in our Patients.

Figure 1 shows the first symptom or onset of this pathology in our patients. However, signs and symptoms most frequently found in the first visit were: Exposed bone (89%), inability to wear prosthesis (70%), suppuration (61%), pain (59%), difficulty eating (52%), halitosis (50%), altered sensation of the jaw (45%), bone loss (41%), loose teeth (34%), mucous ulceration (34%), mucous fistula (32%), tooth loss (23%), skin fistula (11%), oral-sinus communication (11%).

Regarding the biochemical values and hemogram, we must highlight glycemia levels above 110 mg/dL on average in the three groups of patients, and lower hemoglobin (<12.0 g/dL) and hematocrit (<34%) values on average in the group of patients with neoplasia. Serum levels of calcium and phosphorus were normal.

 Table 2 shows the bone remodeling biochemical markers, divided into groups based on their main pathology. We linked lumbar spine, neck and hip total densitome-tric values and ultrasounds of the calcaneus (QUS) to the pathologies, and compared the T-score ( Table 3).

**Table 2:**
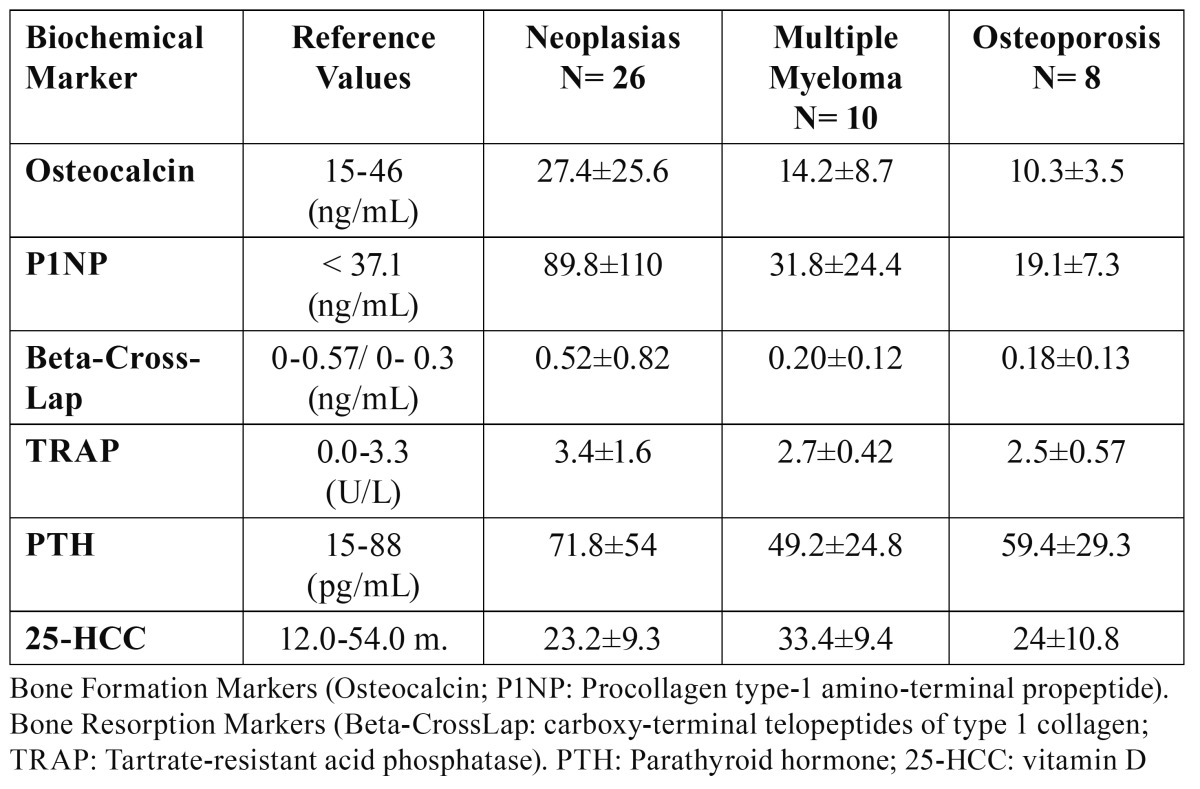
Bone-Remodeling Biochemical Markers, by Pathology.

**Table 3:**
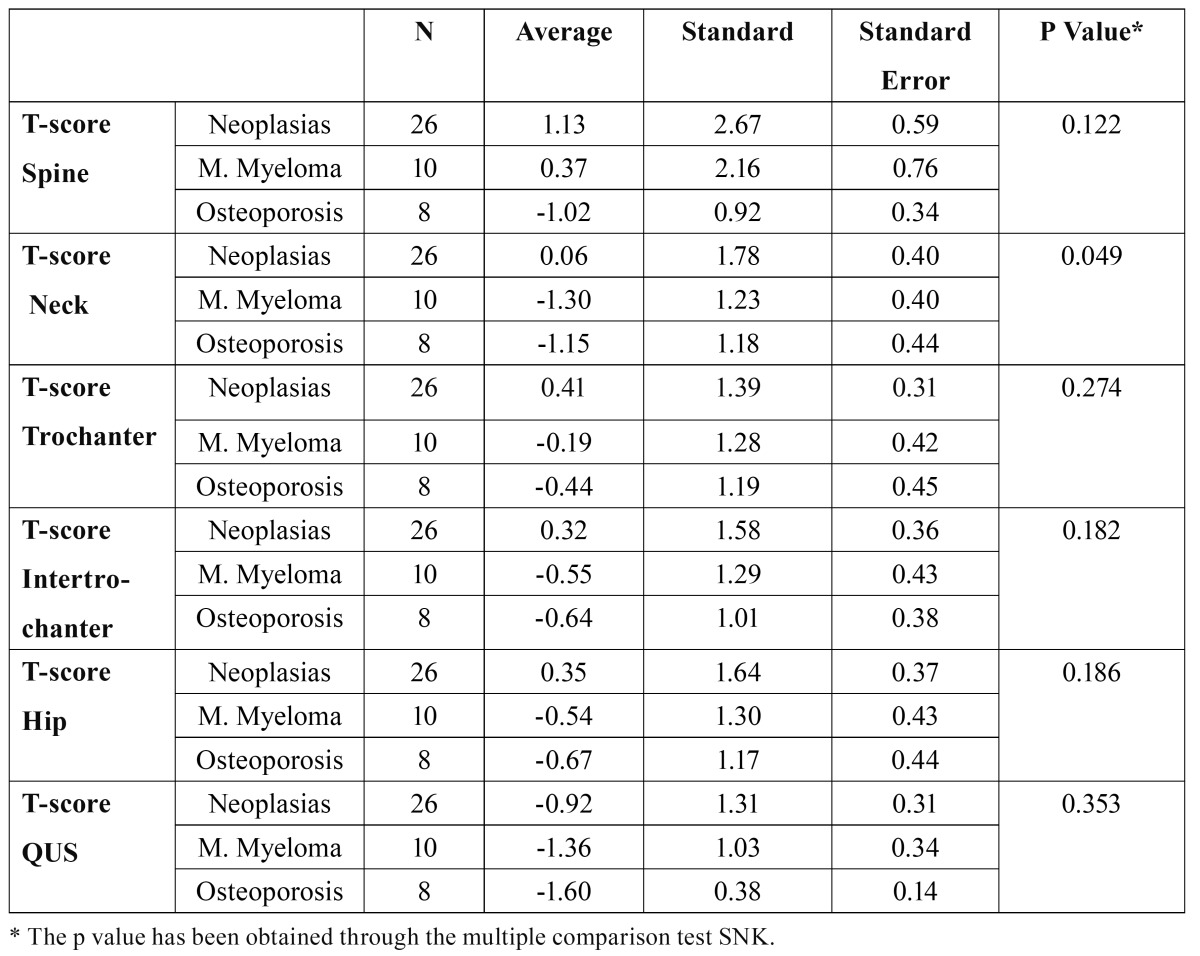
T-score Comparisons per Pathology.

We found pathological vertebral fractures in the spine radiographies of the dorsal-lumbar area in 52% of the cases. Orthopanto-mographies (OPGs) showed radiolucent images in relation to the ONJ lesion, and a diseased alveolar bone in 76% of the cases. The average size of the lesions measured in the 1st-visit OPG was 2.3 cm (1.2–4 cm).

Cultures were taken to perform antibiograms in 45% of the cases. Colonies of Streptococcus mitis and milleri and Gram-negative microorganisms (Enterobacter cloacae, Prevotella spp, Fusobacterium spp) were the most frequently isolated. Biopsies were taken in 32% of the cases for further histological study of the lesions, or as a consequence of the surgical treatments applied. The regular pathological anatomy report was: Oral mucosa with epithelial hyperplasia, fibrosis and chronic inflammation, along with necrotic bone tissue and acute inflammation and many Actinomyces bacterial colonies.

Full bone gammagraphy was undergone by 58% of patients, and an ONJ-compatible pathological captation was found in the upper or lower maxillas in 48% of the cases. The lower jaw gammagraphy was requested for 37% of the patients.

A computed axial tomography (CAT) scan was performed in 34% of the cases to better assess the bone destruction and/or the diseased maxillary sinus.

The therapeutic approach was conservative: cycles of oral antibiotics and 0.12% clorhexidine (CHX) oral antiseptic rinse in all cases. The most frequently used antibiotics were amoxicillin/clavulanic (875/125mg-1000/62.5mg) in 15 to 30-day cycles (92%). Other drugs used (depending on the clinical evolution, allergies or culture results) were: ciprofloxacin (26%), clindamycin (21%), metronidazole (16%), azithromycin (8%), spiramycin (8%) and in some cases: cloxacillin, ampicillin, penicillin, rifampicin, fluconazole. Analgesics were used depending on the pain and the main disease.

Serum, ciprofloxacin and hydrogel (Nu-Gel®) were used as cleansing treatments in 48% of patients. After 2-3 weeks of such treatment, the extra-oral and oral fistulas disappeared and suppurating oral lesions improved in all cases. Local surgical treatment was undergone by 39% of patients, mainly alveolar curettage, and in some cases (8%), sequestrectomy, alveolar segmental osteotomy and mucous flap of the ridge. For 21% of patients presenting small lesions (1.2-3 cm) without extra-oral fistulization, we added the local surgery treatment stra-tegy (sequestrectomy and slight curettage of the underlying bone) and the use of autologous platelet concentrates enriched with growth factors (L-PRP) with the SmartPReP system by Harvest Corp (Plymouth, USA), done bilaterally in the lower jaw, in two cases. These patients improved in 2-4 weeks, their mouth lesions disappeared and they continued to be asymptomatic for an average follow-up period of 14 months.

There is a downward trend in the onset of new ONJ cases in our study. In 2007 we diagnosed 19 cases; in 2008, 11 cases; in 2009, 8 cases; and in 2010, 6 cases. Because of their oncologic diseases, 32% of our patients have passed away, while 45% remain stable with no signs of infection, with or without exposed bone, and 23% still have signs of infection, despite the various pharmacological treatments received.

## Discussion

ONJ is known as a devastating side effect of using BPs in the long-term, although in truth, the genesis of this entity has not been proven yet. Ever since it was des-cribed for the first time in 2003 (8,9), there have been more than 1,000 cases discussed in papers, editorials, letters to the editor and scientific congresses, regarding the strong association between ONJ and BPs (13-17), particularly among patients with MM or solid tumor bone metastases. These patients were generally treated with powerful BPs administered intravenously, but the number of osteoporosis patients with oral BP-related ONJ is increasing (18).

The action mechanism of BPs remains unclear. Being a drug that inhibits bone resorption, used to prevent and treat skeletal complications, why it is related to ONJ remains hard to explain. The fact that the maxillas are the bones subject in the highest degree to bone remodeling, and that they are in direct contact with the mouth’s septic environment, could contribute to it (15).

In our study, the age range is similar to that of other series (19-21). Women are affected the most (64%) in line with the rest of publications (19,22); although men seem to be more affected in some series (20,21).

Zoledronate is the BP associated to ONJ most frequently (20-25). This could be due to the fact that it is a stronger, third generation BP used more and more frequently. Cases of oral BP-related ONJ are also being described in the literature and our own cases (18%)—specifically, alendro-nate, ibandronate, risedronate or clodronate (8,9,18,24-26). Alendronate is the one associated the most, probably because it has been the oral BP prescribed the most.

The most frequently found underlying disease has been breast carcinoma (32%), as in other series (15,19,22). However, MM is more frequent in other studies (23,25).

Regarding the duration of the exposure, it seems the risk of developing ONJ with intravenous BPs starts after 24 months of treatment, and increases after 36 months (21). In our series, the average duration of treatment with zoledronic acid only was 25 months (7-51 months). However, oral BPs need more time and doses, due to their poor bioavailability. Therefore the risk increases after 3 years (4). In our series, the average duration of treatment in alendronate-related cases is 7 years, although in risedronate-related cases it is 26 months, which makes us reflect upon the influence of the drug’s power in the onset of the lesions.

ONJ has been recently described in patients treated with other anti-angiogenic and anti-osteoclastic activity agents similar to BPs, such as bevacizumab and denosumab (27,28). For this reason Stopeck (28) already proposes using a more general term for this pathology: “drug-induced osteonecrosis of the jaws”.

Risk factors or co-factors associated to the physiopathology of the ONJ—besides type, dose, duration of treatment and administration route—are very diverse (5,7). Then, lifestyle-related factors —tobacco, alcohol, inactivity, malnutrition, poor dental hygiene—have been described (11,18,23). In our patients, we have established poor dental hygiene and significant tooth loss, either for lack of motivation or lack of financial resources to have conservative dental treatments done. In agreement with other authors (11,24), we believe there is a potential relationship between ONJ and corticosteroids treatment—in 66% of the cases in our series. In addition, as in some other papers (7,10,11), we believe diabetes to be an important comorbidity factor. The most frequent maxillary location is the lower maxilla (75%), in agreement with the majority of authors. Exodontia prior to developing ONJ is the most important triggering factor known (54.5%); ranging from 38% (15) to 86% (9) in other series. We found exposed bone in the first clinical examination in 89% of the cases, which was an important diagnosis criterion (9,14,18).

Among the bone remodeling biochemical markers, we observe very high osteocalcin and aminoterminal propeptid (P1NP) average values in the group of patients with neoplasia, compared to the group of patients with osteoporosis, which indicates high osteoblastic activity in patients with neoplasia (particularly breast and prostate) possibly related to the bone metastasis. On a different note, suppression of osteoclast activity is not appreciated in these same patients (Beta-CrossLaps, TRAP). The rest of the markers are within the normal ranges.

Regarding bone mineral density, we find very high hip and spine values in oncologic patients, which we suspect could be affected by the baseline condition and the coexisting bone disease (pathological bone in MM, bone metastasis and fractures). The average T-score in the neck is significantly higher in the group of patients with neoplasia (0.06 on average) compared to patients with MM and osteoporosis (p=0.049). However, ultrasound values, which could shed some light on the qua-lity of the bone, actually show no significant differences. The prevalence of vertebral fractures in our series is high (52%), especially in the group of patients with MM (70%), although these patients are treated with very high doses of BPs. We do not have published studies to compare our results.

Managing these patients is very difficult. There are no standard treatments, and patients are weakened by severe oncologic diseases—82% of the cases in our series. We agree with other authors (8,9,11,14,15) in that the conservative or minimally aggressive treatment seems to be the most recommendable course of action. For patients with ONJ and skin fistulas we discussed in a recent paper (29) a conservative treatment based on local cleansing treatments, which is useful, until a definite treatment can be performed. In this series, it is applied to all patients with oral or extra-oral fistulization (48%), which yielded considerable clinical improvement.

Adornato and cols. (30) publish good results by adding to the local surgical treatment a platelet concentrate enriched with growth factors. L-PRP (leukocytes-platelet-rich plasma) was applied in our series. This type of platelet concentrate has potential effects on proliferation, differentiation, immunity and infection, and it favors positive results when treating these lesions surgically.

BPs are not metabolized. They may remain in the bone for a long time after drug suppression. Therefore, some authors (5,15,20) consider that stopping the BP treatment does not seem to avoid the onset or accelerate the reco-very from ONJ . In our series, we have observed clinical improvement in 66% of the cases after stopping the BP treatment, and in oral BP-related cases, 71% showed improvement after 5 months on average (4-7 months), in agreement with other authors (8,9).

Due to the complexity of the treatment, our main goal is prevention and the correct information of patients, and just like other authors (9,10,13-15), we believe prevention and health and dental care coordination are essential in preventing the onset. However, there are no systemic oral or patient indicators so far to predict what patients will develop ONJ due to surgical dental treatments. Possibly, the progressive decline detected in the onset of new cases of ONJ is due to the better understanding of this pathology and the prevention measures adopted, both for patients who are about to start an intravenous BP treatment as for those already receiving it, and also for patients subject to long term oral BP treatments. These patients undergo regular check ups of their oral cavities, their dental and/or prosthesis treatment needs are assessed and we provide them with the guidelines necessary to correctly complete treatments.
